# Symptoms compatible with long COVID in an Italian pediatric cohort of Tourette patients with and without SARS‑CoV‑2 infection: a short-term follow-up assessment

**DOI:** 10.1186/s12887-023-04035-9

**Published:** 2023-05-05

**Authors:** Adriana Prato, Angela Maria Salerno, Federica Saia, Nicoletta Maugeri, Alice Zanini, Miriam Scerbo, Rita Barone, Renata Rizzo

**Affiliations:** Child and Adolescent Neurology and Psychiatric Section, Department of Clinical and Experimental Medicine, Catania University, Catania, 95124 Italy

**Keywords:** Tourette Syndrome, COVID-19, Long COVID, Pandemic, Mental health, Children, Pediatrics

## Abstract

**Background:**

Tourette Syndrome (TS) is a childhood-onset neurodevelopmental disorder with a worldwide prevalence of about 0.3–1% of the population. During the pandemic caused by SARS-CoV-2 infection, the impact on the mental health of children and adolescents was very important. The persistence of symptoms in the post-acute phase of the disease has been termed Long COVID. The neuropsychiatric symptoms seem to be the most common impairment in children and adolescents with long COVID.

**Objectives:**

Considering the impact of pandemic on mental health, in this study we analyzed the long-term effects of SARS-CoV-2 infection in children and adolescents affected by TS.

**Methods:**

We conducted an online questionnaire covering socio-demographic and clinical data among 158 patients affected by TS or chronic tic disorders (CTD), of which 78 participants reported a positive SARS-CoV-2 infection. Data were collected to investigate tic severity and both the comorbidities, as well as lockdown-related changes to daily life activities and, in case of infection of SARS-CoV-2, possible symptoms of acute infection and long COVID. Markers of systemic inflammation including C-reactive protein (CRP), erythrocyte sedimentation rate (ESR), ferritin, iron, electrolytes, white blood cell counts, platelet cell counts levels, markers of liver, kidney and thyroid function were analyzed. First, all patients were screened with the Schedule for affective disorders and Schizophrenia for School age children—present and lifetime (Kiddie-SADS-PL) to rule out primary psychiatric disorders considered as criteria of exclusion. Then, all patients were clinically assessed at baseline (T0), and after three months (T1) through the administration of Yale Global Tic Severity Rating Scale (YGTSS), Multidimensional Anxiety Scale for Children (MASC), Child Depression Inventory (CDI) and Child Behavior Checklist (CBCL).

**Results:**

Among the cohort of TS patients that contracted SARS-CoV-2 infection, 84.6% (*n* = 66) experienced any acute symptoms, and long COVID symptoms occurred in 38.5% (*n* = 30). A worsening of clinical symptoms of tics and eventually associated comorbidities occurred in 34.6% (*n* = 27) of TS patients that contracted SARS-CoV-2 infection. TS patients with or without SARS-CoV-2 infection showed an increase in the severity of tics and also behavioral, depressive and anxious symptoms. Instead, this increase was more evident in patients who contracted the infection than in patients who did not contract it.

**Conclusions:**

SARS-CoV-2 infection may have a role in the increase of tics and associated comorbidities in TS patients. Despite of these preliminary results, further investigations are necessary to improve knowledge about the acute and long-term impact of SARS-CoV-2 in TS patients.

**Supplementary Information:**

The online version contains supplementary material available at 10.1186/s12887-023-04035-9.

## Background

Tourette syndrome (TS), also known as Gilles de la Tourette syndrome or Tourette disorder, is a complex, childhood-onset neuropsychiatric condition that includes multiple motor and vocal tics with duration of at least 1 year [3]. Children with TS frequently present other neuropsychiatric conditions in comorbidity, such as attention deficit hyperactivity disorder (ADHD), obsessive–compulsive disorder (OCD), anxiety disorder or oppositional defiant disorder (ODD) [17]. The etiology of TS and tic disorders is complex and multifactorial, in which genetic, immunological and hormonal factors interact to establish vulnerability. Several immune-mediated mechanisms, including microglial dysfunction, reduced numbers of regulatory T cells, altered gamma globulin, and an increased response to pathogens, have been proposed in the pathogenetic mechanism of TS and related neuropsychiatric disorders [38]. In children, the risk of severe acute respiratory syndrome coronavirus 2 (SARS-CoV-2) being severe is low. Furthermore, children infected with SARS‑CoV‑2 are often asymptomatic or have mild symptoms with very rare hospitalization or death [46]. The two main long-term complications of SARS-CoV-2 infection are Multisystem Inflammatory Syndrome in Children (MIS-C), an acute and potentially fatal condition that occurs 2 to 6 weeks after SARS-CoV-2 infection in less than 0.1% of pediatric cases, and long COVID [43]. The persistence of symptoms in the post-acute phase of the disease has been termed long COVID. Researchers have utilized the best available evidence using a modified Delphi process to define long COVID that occurs in young people with a history of confirmed SARS-CoV-2 infection with at least one persisting physical symptom for a minimum duration of 12 weeks after initial testing that cannot be explained by an alternative diagnosis [40]. Despite of adults, long COVID has been poorly studied in children. In fact, a very low number of studies concerning the long-term effects of SARS-CoV-2 infection in the first years of age has been conducted. Furthermore, the results of the available studies are often conflicting, and this does not help to acquire convincing data on pediatric long COVID [12]. The clinical manifestations of long COVID are highly variable in symptoms, intensity, and duration. One of the most common symptoms found for children is constant fatigue, reported in up to 87% of patients [13]. Other symptoms include headache, sleep disturbances, difficulty concentrating, abdominal pain, myalgia or arthralgia, persistent chest pain, stomach pain, diarrhea, heart palpitations and skin lesions. These clinical manifestations can occur both alone and in combination, can be transient or intermittent, can change over time or remain constant. Although long COVID manifestations seem to be more common in children with symptomatic or severe acute SARS-CoV-2 infection, they are also described in asymptomatic or paucisymptomatic patients [6]. The neuropsychiatric symptoms seem to be the most common impairment in children and adolescents with long COVID. While the prognosis of children with long COVID is generally good, some children may develop long-term symptoms that have a profound impact on daily family life [14].

The most frequent neuropsychiatric symptoms include fatigue, post-exertional malaise (PEM), cognitive dysfunction, headache, sleep disturbances, behavioral symptoms [37]. Several studies carried out during previous severe epidemics have also shown that, together with medical problems, children could have severe psychological repercussions, with the development of frustration, worry or sadness, and feeling of being alone and being excluded by family or community [25]. During the pandemic, the impact on the mental health of children and adolescents was also greater. A major role in the development of mental disturbances has been played by the measures put in place worldwide by health authorities to reduce viral circulation [12]. Mental health of children has been severely compromised on account of increased anxiety, changes in their diets, school dynamics and education, fear of not knowing how to deal with emerging problems [39]. Development of mental health disfunctions was found more common in older children and adolescents, in females and in those with previously diagnosed psychological problems. This is quite in agreement with what has been reported for pediatric long COVID, further suggesting that most of the clinical manifestations characterizing long COVID depend on the pandemic and not directly on the infection [12]. Considering the impact of pandemic on mental health, in this study we present self-reported data from a study on children and adolescents affected by TS with and without SARS-CoV-2 infection. We sought to provide an overview of their clinical symptoms and determine whether symptom variations were associated with specific clinical variable. Specifically, we performed a through evaluation to (1) examine clinical differences between patients with and without SARS-CoV-2 infection, (2) assess and evaluate outcome of tics and associated comorbidities both in TS patients with SARS-CoV-2 infection and in patients without SARS-CoV-2 infection, and (3) evaluate the long-term effects of SARS-CoV-2 infection in paediatric patients affected by TS.

## Materials and methods

### Study design

This study was conducted at the Child and Adolescent Neurology and Psychiatry of the Medical and Experimental Department of Catania University, Italy. A total of 158 patients (117 boys and 41 girls) with a diagnosis of TS or Chronic Tic Disorder (CTD), according to the Diagnostic and Statistical Manual for Mental Disorders (DSM-V), have been enrolled. All patients with tic disorders seen between January 2022 and May 2022 were investigated for SARS-CoV-2 infection positivity, reported using rapid antigen tests, and in uncertain cases confirmed through molecular tests. Patients were recruited among a clinical population of children and adolescents with tic disorders admitted at the outpatient clinic in the study period. Based on the antigen test’s results, participants were assigned into two groups, the “COVID + group” (*n* = 78), which resulted positive for SARS-CoV-2 infection, and the “COVID- group” (*n* = 80), which resulted negative for SARS-CoV-2 infection.

### Participants

Eligible participants were patients aged 3–18 years of age with a primary diagnosis of TS or CTD according to DSM-5 criteria, recruited from January 2022 to May 2022 at the continuous outpatient clinic of the Child and Adolescent Neuropsychiatry Unit at Catania University Hospital. We excluded patients affected by other primary psychiatric disorders different from TS or CTD, and children where was started or adjusted any psychotropic medication for tic within the previous two months. Before inclusion in the study, all patients were screened with the Schedule for affective disorders and Schizophrenia for School age children—present and lifetime (Kiddie-SADS-PL) to rule out primary psychiatric disorders considered as criteria of exclusion. Then, all patients underwent neuropsychiatric evaluation for TS and related comorbidities. The Kiddie-SADS-PL is a semi-structured interview tool developed by Kaufman et al. [19] that can be used in children and adolescents aged between 6–18 years [19]. Each participant was clinically assessed for cognitive abilities, TS symptoms and associated comorbidities. Fasting blood samples were drawn to analyze laboratory markers of systemic inflammation including C-reactive protein (CRP), erythrocyte sedimentation rate (ESR), ferritin, iron, electrolytes, white blood cell counts, platelet cell counts levels, and also markers of liver, kidney and thyroid function. All the procedures adopted for the current research were performed as part of the routine clinical assessment. Written informed consent was obtained from all TS participants’ parent, caregiver or legal guardian to enter clinical data from the clinical files into the present study.

### Data source

For data collection, we developed an online questionnaire covering socio-demographic and clinical data including tic severity and both the comorbidities, as well as lockdown-related changes to daily life activities and, in case of infection of SARS-CoV-2, possible symptoms of acute infection and long COVID (Figure S1). The questionnaire, administered at T0 to the parents, caregivers or legal guardians of the patients, was directed to a clinical sample of children and adolescents with a DSM-5 validated diagnosis of TS or CTD. Participant’s parents, caregivers or legal guardians were asked to report on tics and eventually variations on symptoms, eventual change in the pharmacological approach, as well as if their children had been vaccinated against SARS-CoV-2 infection and experienced any side effects. Furthermore, a survey section was dedicated to the TS patients that contracted SARS-CoV-2 infection. For these patients, we asked whether they experienced any acute symptoms of infection, what medication they used to treat the infection, and if they showed symptoms of long COVID. All participant’s parents, caregivers or legal guardians gave informed consent before filling their questionnaire anonymously.

### Clinical assessment

The clinical assessment of the patients was performed at two time points during the study by pediatric neuropsychiatrists with solid experience in tic disorders and possible comorbidities. Participants underwent the first assessment at baseline (T0), the second assessment after three months (T1). At T0, the Wechsler Intelligence Scale for Children (WISC-IV) was administered to evaluate the IQ of patients [44]. At baseline point (T0), patients were also assessed according to Yale Global Tic Severity Rating Scale (YGTSS), Multidimensional Anxiety Scale for Children (MASC), Child Depression Inventory (CDI) and Child Behavior Checklist (CBCL). After two months (T1), changes in symptoms severity were evaluated by improvement in the YGTSS, MASC, CDI and CBCL scales. The YGTSS is a clinician-rated scale used to assess the motor and phonic tic severity considering the number, frequency, duration, intensity, and complexity of tics. It consists of separate motor and vocal tic checklists scored from 0 to 5 on two subscales for motor and vocal tics. The subscales were combined to produce a total tic severity score (ranging from 0 to 50). Another score ranging from 0 to 50 was assigned for global impairment due to tics [22]. The CBCL is a caregiver completed questionnaire of child behavioral and emotional problems which is standardized, objective, and widely utilized by child psychiatrists, pediatricians, developmental psychologists, and other mental health professionals for clinical and research purposes [1]. Finally, all participants completed the MASC, a self-report scale that robustly represents the factor structure of anxiety in children aged 8–18 years [26] and the CDI, a 27-item self-report instrument that assesses depressive symptoms in 7- to 17-year-olds [20].

### Statistical analysis

Data were analyzed using SPSS software (SPSS, Inc., Chicago, IL, USA, IBM, Somers, NY, USA). Demographic characteristics of participants and their clinical outcomes at baseline and 2 months were summarized using mean (SD) for continuous data or count (%) for categorical data. We assessed the distribution of quantitative variables to determine their deviation from the normal distribution (Shapiro–Wilk test). Students’ t-tests were used to compare the neuropsychological scores and clinical characteristics between two groups. Clinical outcomes among T0 and T1 were also evaluated to identify patients who showed a worsening of clinical symptoms. A *p*-value < 0.05 was considered to indicate statistical significance.

## Results

### Sample description

We enrolled a total of 158 patients aged 3–18 years (mean age = 10.9 ± 3.6; male (M)/female (F) = 117:41; male = 74.1%). All patients were affected by TS or CTD. Only 89 patients (56.3%) presented “pure-TS” phenotype. The mean age of tic onset was 6,9 ± 2,4 years. Regarding treatment approaches in TS/CTD, 38 patients had received cognitive behaviour therapy (CBT) (24.05%), and 53 patients take anti-tic medications (33.5%) including atypical antipsychotics (such as risperidone, aripiprazole), alpha2-adrenergic agonists or other pharmacological categories for TS treatment. 70 participants (44.3%) had been vaccinated against SARS-CoV-2 infection, and 18 patients of them (11.4%) reported any side effects, most frequently arm pain (*n* = 12) and fever (*n* = 5).

Seventy-eight participants reported at baseline report a positive SARS-CoV-2 infection (49.4%). No statistically significant differences were observed based on demographic and clinical features in the COVID + group vs. COVID- group, except for age of tic onset (Table 1). Compared with TS patients of COVID- group, TS patients of COVID + group were younger at symptom onset (mean age = 6.5 ± 2.4 vs 7.2 ± 2.3, *p* = 0.03).

**Table 1:** Participant features

**Participant characteristics**	**COVID + group** **(*n* = 78)**	**COVID- group** **(*n* = 80)**	***P*-value**
**Male (%)**	55 (70.5%)	62 (77.5%)	0.32
**Age**	10.8 (± 3.7)	11.05 (± 3.5)	0.64
**Age of onset**	6.5 (± 2.4)	7.2 (± 2.3)	**0.03**
**Comorbid diagnosis (yes, %)**	40 (51.3%)	49 (61.25%)	0.21
**TS-Only**	38 (48.7%)	31 (38.75%)	0.21
**TS + ID/DD**	8 (10.3%)	12 (15%)	0.37
**TS + OCD**	15 (19.2%)	13 (16.25%)	0.62
**TS + CD**	16 (20.5%)	13 (16.25%)	0.49
**TS + ASD**	3 (3.8%)	6 (7.5%)	0.32
**TS + LD**	3 (3.8%)	5 (6.25%)	0.49
**TS + Others**	12 (15.4%)	23 (28.75%)	**0.04**
**Pharmacological Treatment**	30 (38.5%)	23 (28.75%)	0.19
**Behaviour therapy**	20 (25.6%)	18 (22.5%)	0.64
**COVID vaccination**	30 (38.5%)	41 (51.25%)	0.11
**Side effects**	8 (10.3%)	9 (11.25%)	0.84

**Fig. 1:**
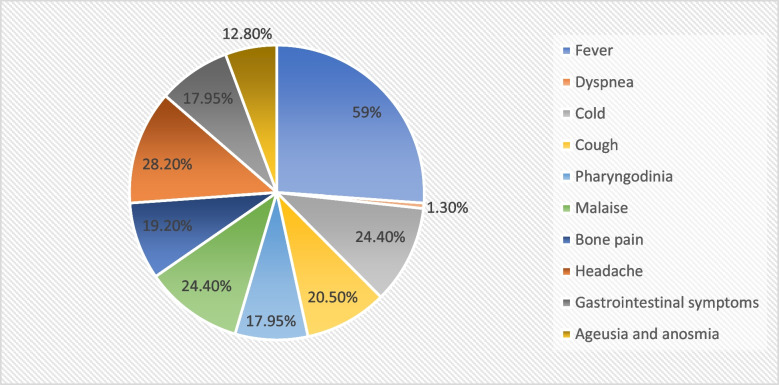
Presence of symptoms in TS patients during SARS-CoV-2 infection

*TS* Tourette syndrome, *ID* Intellectual disability, *DD* Developmental delay, *OCD* Obsessive–compulsive disorder, *CD* Conduct disorder, *ASD* Autism spectrum disorder, *LD* Language disorder;

Among the COVID + group,, 84.6% (*n* = 66) experienced any acute symptoms of infection, most frequently fever (59.0%, *n* = 46), cold (24.4%, *n* = 19), malaise (24.4%, *n* = 19) and headache (28.2%, *n* = 22) (Fig. 1).

**Fig. 2:**
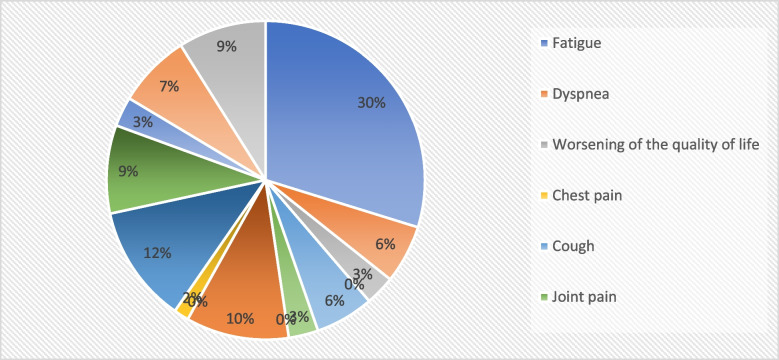
Long COVID symptoms in TS patients with SARS-CoV-2 infection

Mean disease duration was 3.7 (± 3.7) days. Furthermore, 53.8% (*n* = 42) of them used pharmacological treatment for the infection, but only in 1 patient (1.3%) would be necessary a variation of the pharmacological treatment. Instead, a worsening of clinical symptoms of tics and eventually associated comorbidities occurred in 34.6% (*n* = 27) of TS patients of COVID + group. Long COVID symptoms occurred in 38.5% (*n* = 30), most frequently fatigue (25.6%, *n* = 20), headache (9%, *n* = 7), difficulty in completing daily activities (10.3%, *n* = 8), attention deficit (7.7%, *n* = 6) and sleepiness (7.7%, *n* = 6) (Fig. 2).

As to routine laboratory blood analyses conducted in 140/158 TS patients (88.6%), we found no significant differences among the two study groups when comparing CRP (*p* = 0.193), ESR (*p* = 0.925), ferritin (*p* = 0.784), iron (*p* = 0.162), white blood cell counts (*p* = 0.903), platelet cell counts levels (*p* = 0.707), and also markers of liver (*p* = 0.652), kidney (*p* = 0.124) and thyroid function (*p* = 0.298). Electrolytes level’s adnormalities tended to be more representative in the COVID + group (*n* = 45, 66.2%) compared with COVID- group (*n* = 34, 47.2%) (*p* = 0.024).

### Baseline characteristics

At baseline, no statistically significant differences were observed based on neuropsychological findings in COVID + group vs. COVID- group. The mean scores for YGTSS, CBCL, MASC and CDI were slightly higher in COVID + group vs. COVID- group (YGTSS: mean 18.0, SD 7.75 vs. mean 17.1, SD 7.4, *p* = 0.44; CBCL: mean 28.0, SD 20.4 vs. mean 24.1, SD 18.96, *p* = 0.21; MASC: mean 22.6, SD 22.9 vs. mean 19.1, SD 22.0, *p* = 0.17; CDI: mean 4.1, SD 6.6 vs. mean 3.2, SD 5.0, *p* = 0.18).

### Yale Global Tic Severity Rating Scale (YGTSS) outcome

**Fig. 3:**
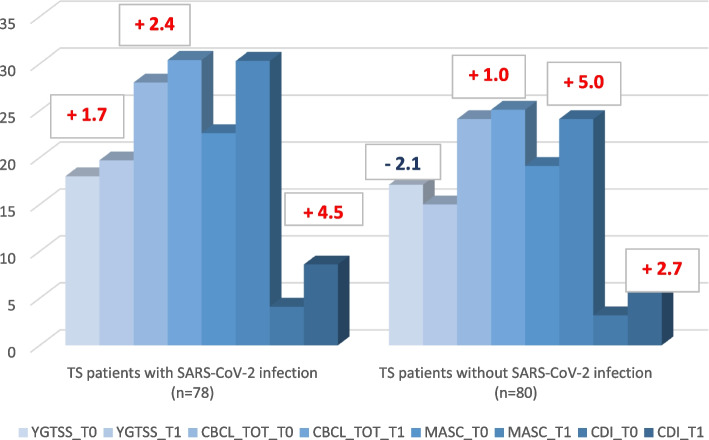
Yale Global Tic Severity Rating Scale (YGTSS), Child Behavior Checklist (CBCL), Multidimensional Anxiety Scale for Children (MASC), Child Depression Inventory (CDI) outcome in TS patients with or without SARS-CoV-2 infection

In general, TS patients of COVID + group showed an increase in the severity of tic symptoms, as assessed by YGTSS scores and sub-scores, at T1. Mean YGTSS score at 2 months after the infection was 19.7 (SD 9.6), with a mean total increase of 1.7. Conversely, in the COVID- group, patients showed a reduction in the severity of tic symptoms, as assessed by YGTSS scores and sub-scores, at T1 (mean YGTSS score = 15.0, SD 7.3), with a mean total decrease in YGTSS at 3 months of 2.1. The variations in YGTSS scores are shown in Fig. 3.

### Child Behavior Checklist (CBCL) outcome

Both groups showed an increase in the severity of behavioral symptoms, as assessed by CBCL scores and sub-scores, at T1. Mean total CBCL score at T1 was 30.4 (SD 21.8) in the COVID + group, compared with 25.1 (SD 18.7) in the COVID- group. The mean total increase in CBCL at 3 months was 2.4 in the COVID + group vs. COVID- group). The variations in CBCL scores are shown in Fig. 3.

### Multidimensional Anxiety Scale for Children (MASC) outcome

Patients including in both groups showed an increase in the severity of anxious symptoms, as assessed by MASC scores, at T1. Mean total MASC score at T1 was 30.3 (SD 21.65) in the COVID + group, compared with 24.1 (SD 21.8) in the COVID- group. The mean total increase in MASC at 3 months was 7.7 in the COVID + group versus 5.0 in the COVID- group (Fig. 3).

### Child Depression Inventory (CDI) outcome

TS participants of both groups showed an increase in the severity of depressive symptoms, as assessed by CDI scores, at T1. Mean total CDI score at T1 was 8.6 (SD 6.4) in the COVID + group, compared with 5.9 (SD 5.6) in the COVID- group. The mean total increase in CDI at 3 months was 4.5 in the COVID + group versus 2.7 in the COVID- group. The variations in CDI scores are shown in Fig. 3.

## Discussion

Several studies have already showed that the COVID pandemic have a significant impact on the mental health of young people with TS, worsening both tics and emotional and behavioral symptoms. The social contexts for children and young people during this last year have been markedly different to what they had experienced before [15, 30, 34] did not observed significant differences in tic symptom or severity between participants who were assessed before and during the pandemic [15]. Conversely, since the onset of the COVID19 pandemic, it was reported an increase in tic symptoms in some children and adolescents who already had a diagnosis of a tic disorder [8, 16] In a recent study, self-reported data on the impact of lockdown in school-age patients with tic disorders indicate perceived changes in tic severity, as well as restlessness and irritability, in about half of the cases [41]. A worsening of tics in patients affected by TS, after the lockdown, has not been observed only in pediatric patients, but also in adults with TS/CTD [10]. The increase and worsening of tics during the pandemic can be explained by the evidence that, psychosocial stress is largely implicated in the severity and frequency of tics [7]. Considering the general abrupt growth in neuropsychiatric symptoms during the recent pandemic period, it was possible to hypothesize that the worsening of tics and associated comorbid symptoms is strongly related to lockdown measures, the psychological pressure of the pandemic, and the major vulnerability to emotional events in children and adolescents. Neurological and psychiatric symptoms of long COVID have been reported even in patients without a previous diagnosis of tic disorders. Abnormal movements, anxiety, emotional dysregulation and other neuropsychiatric symptoms were reported in a case series of five children from a few weeks to months after the resolution of the acute infection [36]. Pavone et al. [29] also reported on two unrelated children with new-onset paediatric acute onset neuropsychiatric syndroms (PANS) that started two weeks after a positive COVID-19 infection [29]. Our results showed a more significant worsening of tics in TS patients that contracted SARS-CoV-2 infection, compared with TS patients without SARS-CoV-2 infection. This was supported by an increased score at YGTSS, that confirmed the data obtained from the administered questionnaire: TS patients with SARS-CoV-2 infection presented a mean YGTSS score at 3 months post-infection of 19.5 (SD 9.8), with a mean total increase of 1.3; in contrast, in the TS cohort without SARS-CoV-2 infection, patients showed a reduction in tic symptom severity, as assessed by YGTSS scores, at T1 (mean YGTSS score = 15.4, SD 7.35), with a mean total decrease in YGTSS at 3 months of 1.9. In general, patients of both groups reported an increase non only of tics but also of comorbid symptoms. The worsening of symptoms observed was more severe in patients of COVID + group compared on patients of COVID- group. Moreover, we can speculate that the decrease of tic symptoms observed at follow-up visit in patients without SARS-CoV-2 infection may be probably attributable to the minor severity of tics at baseline, and to the minor presence of other associated comorbidities that further complicate their clinical course., Long COVID symptoms occurred in 36.2% (*n* = 25) of patients of COVID + group:, the most experienced symptom was fatigue in 21.7% (*n* = 15), followed by headache and difficulty in completing daily activities both in 10.1% (*n* = 7). To the best of our knowledge, no studies have been already focused on long COVID effects on patients with tic disorders. In general, children were less likely to develop long COVID when compared to adults [42]. Instead, in a report conducted by Asadi‑Pooya et al. [4], twenty-six (44·8%) children/adolescents reported symptoms/complaints of long COVID, including fatigue (21%), shortness of breath (12%), exercise intolerance (12%), weakness (10%), and walking intolerance (9%). In our study, we assessed tics, but also co-occurring conditions, including anxious and depressive symptoms, and behavioral disorders. Other literature studies conducted on paediatric samples observed similar results. Pasca et al. [28] reported increased scores in CBCL questionnaire during the global pandemic caused by SARS-CoV-2 infection in pediatric patients with neuropsychiatric disorders. In another study, 25% of families reported during home quarantine the exacerbation of some behavioral problems, such as more frequent and intense episodes of non-collaboration, indifference, physical/verbal aggression, poorly targeted/organized play, screaming/crying, social iso- lation, provocative attitudes towards others, attempts to escape, and self-cutting ideation, in line with our study [33]. In a meta-analysis conducted on 80,879 children, the pooled prevalence of depression and anxiety symptoms during pandemic has doubled compared to prepandemic estimates [32]. These findings are consistent with previous studies that have shown that compared to adults, children and adolescents are at higher risk for depression and anxiety after natural disasters [9, 42]. In our study, no statistically significant differences were observed between both groups in CBCL scores at T1: in fact, the mean score in the first group was 31.1 (SD = 22. 2), while in the second is 25 (SD = 18.4). As these scores showed, in both groups we observed an increase in the severity of behavioral, depressive and anxious symptoms: this increase, however, was more important in patients who contracted SARS-CoV-2 infection than in patients who did not contract it.

The underlying cellular and molecular mechanisms underlying the various clinical spectrum of long COVID are poorly understood. The proposed pathophysiological mechanisms for the development of long COVID seems to be include: hyperinflammatory state, chronic immune activation, renin-angiotensin system dysfunction leading to central and peripheral circulatory abnormalities, mast cell activation, virus persistence and organ damage, interference with fibrinolysis and promotion of micro-thrombi, and the development of auto-antibodies [5, 27]. Lastly, infections or post-infections autoimmune effects-post-SARS-CoV-2 might play an important role [40, 45]. The increase of tics observed in TS patients with SARS-CoV-2 infection could also in part be related to the virus pathogenesis: SARS-CoV-2 is directly neuro-invasive and causes a cytokine firestorm with consequences on the central nervous system (CNS); as a consequencee, cognitive and psychiatric follow-up of these patients will certainly be detected specifically in future. The altered immunological response seems to play a predominant role. Recently, the immunological differences between children recovered from the SARS-CoV-2 acute infection and children with post-acute sequelae (PASC) have been detected, including the possible predominant role of the innate immune system [11]. Therefore, in the alveoli, after the initial damage to the blood–lung barrier, chronic inflammation with continuous production of pro-inflammator cytokines and reactive oxygen species (ROS) may occur, followed by their release into surrounding tissues and bloodstream [13]. Abnormalities in coagulation can also increase the risk of microthrombosis in multiple organs but especially in alveolar capillaries, with an increased risk of thrombotic events. The blood–brain barrier dysregulation can then allow cytokines and leukocytes to infiltrate the brain parenchyma. Following SARS-CoV-2 infection, a “cytokine storm” from effector cells such as interleukin (IL)-6, IL-2, IL-17, interferons (IFN)-alpha, IFN-gamma, tumor necrosis factor (TNF)-alpha, induces a neuroinflammatory response causing disruption of the blood–brain barrier (BBB), leading to peripheral immune cell transmigration into the brain and, in turn, causes imbalances in neurotransmission [35]. Moreover, several studies suggested that microbiome/virome dysbiosis can impact initial SARS-CoV-2 risk and virulence, highlighting the importance of microbiome for the maintenance of symptoms [31]. All these pathophysiological mechanisms can lead to the possible and various neurological manifestations recognized in patients with long COVID. Overall, patients affected by tic disorders shown possible abnormalities in the development of immune responses. Furthermore, a conspicuous number of studies have displayed hyperactive systematic immune-inflammatory responses in TS patients [24, 18]. Post-mortem transcriptome analyses of the striatum of adults patients with TS compared with brain tissue from gender and age matched control group [23] detected 309 downregulated genes, 822 upregulated genes, and 17 gene coexpression modules associated with TS. The top-scoring upregulated module included immune-related genes, consistent with these author’s observations [23]. Recently, [2] have investigated immune and inflammatory pathways in post-mortem brain tissue of individuals with Autism Spectrum Disorders (ASD) and TS and they reported enriched pathways involving inflammation, cytokines, signal transduction and cell signalling in ASD and TS brain transcriptome [2]. Instead, our results confirms the risk of persistent symptoms in paediatric patients with long COVID, and highlight the importance of a long-term follow-up.

The current study has several limitations. First, our study had a short follow-up period, so an observation period longer than 3 months may be needed to show the onset of other symptoms of long COVID and worsening or improvement of the underlying pathology. Furthermore, considering the temporal pattern of tics, which are known to occur in bouts and wax and wane in severity [21], a longer follow-up observation might represent important clues to the course and phenomenology of tics in our sample. Whether the increase of tics and associated symptoms observed in our sample of children with long COVID are the consequence of SARS-CoV-2 infection or are due to the tremendous impact resulting from the social isolation rules and lockdowns is still not clear. Second, our study did not include a non-TS control group infected by SARS-CoV-2. Considering these limitations, the results should be considered as preliminary rather than conclusive and may lay the groundwork for subsequent studies. On the other hand, this study also had several strengths, including carefully considered inclusion and exclusion criteria, the assessment of not only tics but also co-occurring conditions, the inclusion of a control group of children with TS without SARS-CoV-2 infection, and also the observation of long COVID symptoms. In conclusion, our results suggest that there was a significant increase in tics and also behavioral, depressive and anxious symptoms in TS patients with SARS-CoV-2 infection, compared with TS patients without the infection; in addition, long COVID symptoms were also observed in the infected patients.

## Conclusion

This study shows that SARS-CoV-2 infection may have a role in the increase of tics and associated comorbidities in TS patients, even if our data are preliminary and further investigations are necessary. Despite of these results, further studies are needed to better understand the the impact of SARS-CoV-2 in TS patients, not limited only to the assessment of the acute phase of the infection, but also evaluating the persistence of long COVID symptoms. In conclusion, more knowledge is needed to recognize the true prevalence of long COVID in children, better understand the long-term consequences of SARS-CoV-2 infection in TS patients, and also improve their management.

## Supplementary Information

**Additional file 1: Supplementary Figure 1.** Online Questionnaire.

## Data Availability

The data that support the findings of this study are available from the corresponding author upon reasonable request.

## Abbreviations

ADHD: Attention Deficit Hyperactivity Disorder
CBCL: Child Behavior Checklist
CBT: Cognitive behaviour therapy
CDI: Child depression inventory
CNS: Central Nervous System
CRP: C-reactive protein
CTD: Chronic Tic Disorder
DSM-V: Diagnostic and Statistical Manual for Mental Disorders
ESR: Erythrocyte sedimentation rate
Kiddie-SADS-PL: Schedule for affective disorders and Schizophrenia for School age children—present and lifetime
MASC: Multidimensional Anxiety Scale for Children
MIS-C: Multisystem Inflammatory Syndrome in Children
OCD: Obsessive–Compulsive Disorder
ODD: Oppositional Defiant Disorder
PEM: Post-Exertional Malaise
SARS-CoV-2: Severe Acute Respiratory Syndrome Coronavirus 2
TS: Tourette Syndrome
WISC-IV: Wechsler Intelligence Scale for Children
YGTSS: Yale Global Tic Severity Rating Scale
