# Supplementary material for: Symptoms compatible with long COVID in an Italian pediatric cohort of Tourette patients with and without SARS‑CoV‑2 infection: a short-term follow-up assessment
Source: BMC Pediatr. 2023 May 5;23:222. doi: 10.1186/s12887-023-04035-9 (PMC10161986; doi:10.1186/s12887-023-04035-9)
Supplement: Supplementary file 1 — Additional file 1: Supplementary Figure 1. Online Questionnaire. [file 12887_2023_4035_MOESM1_ESM.docx]

**Supplementary Figure 1. Online Questionnaire**

|  | **YES** | **NO** | **OPEN ANSWERS** |
| --- | --- | --- | --- |
| Has your child ever contracted SARS-CoV-2 infection? |  |  |  |
| When he/she contracted the infection? |  |  | Date / _/__ |
| Was he/she asymptomatic or did he/she present few symptoms? |  |  | Asyntomatic 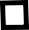  Paucisyntomatic 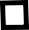  Symptomatic 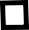 |
| If with few symptoms, what symptoms did he present? |  |  | - Fever 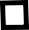 - Shortness of breath 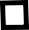 - Cold 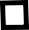 - Cough 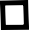 - Sore throat 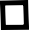 - Illness 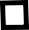 - Bone aches and pains 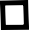 - Headache 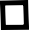 - Enteric symptoms 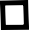 - Ageusia and Anosmia 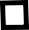 |
| Have other family members contracted SARS-CoV-2 infection? |  |  |  |
| How long were the symptoms? |  |  | No. Of days: ______ |
| Has the child taken medication to treat the infection? |  |  |  |
| Did the child have to suspend or change the treatment of the underlying disease? |  |  |  |
| After how many days has the child recovered? |  |  | No. Of days: |
| Has the underlying condition worsened/increased? |  |  |  |
| Have you noticed any new symptoms? |  |  |  |
| Did you detect any persistence of SARS-CoV-2 infection’s symptoms in the child after recovery?  (If yes, please indicate which of the listed symptoms) |  |  | - Fatigue 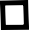 - Dyspnea 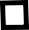 - Worsen of quality of life 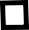 - Chest pain 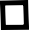 - Cough 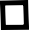 - Joint pain 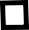 - Palpitations 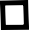 - Headache 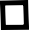 - Skin rashes 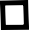 - Enteric symptoms 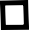 - Inabililty to do routine daily activities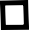 - Attention disorder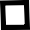 - Hair loss 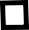 - Sleepiness 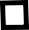   Sleep disorders 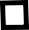 |
| Has the child been vaccinated?  If yes, please indicate if first dose/second dose/booster dose |  |  | - First dose 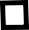 - Second dose 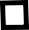   Booster dose 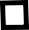 |
| Did the child have any side effects to the vaccine? |  |  |  |
| When was the last dose? |  |  | Date_ / _/ _ |
